# Cell-SELEX Aptamer for Highly Specific Radionuclide Molecular Imaging of Glioblastoma *In Vivo*

**DOI:** 10.1371/journal.pone.0090752

**Published:** 2014-03-06

**Authors:** Xidong Wu, Huiyu Liang, Yan Tan, Chao Yuan, Shuji Li, Xiaowen Li, Guiping Li, Yusheng Shi, Xingmei Zhang

**Affiliations:** 1 Department of Neurobiology, School of Basic Medical Sciences, Southern Medical University, Guangzhou, China; 2 Department of Neurology, Nanfang Hospital, Southern Medical University, Guangzhou, China; 3 Department of Nuclear Medicine, Nanfang Hospital, Southern Medical University, Guangzhou, China; 4 Department of Radiation Oncology, Nanfang Hospital, Southern Medical University, Guangzhou, China; Wake Forest University, School of Medicine, United States of America

## Abstract

Glioblastoma (GBM) is the most frequent and aggressive primary adult brain tumor with poor prognosis. Epidermal growth factor receptor variant III (EGFRvIII) is the most common and highly oncogenic EGFR mutant in GBM. With the aim to generate specific molecular probes able to target EGFRvIII with high affinity, we selected four DNA aptamers (U2, U8, U19 and U31) specifically bound to U87-EGFRvIII cells that over expressed EGFRvIII with *K*_d_ values in the nanomole range by a cell-based systematic evolution of ligands by exponential enrichment (cell-SELEX) process. U87MG cells were introduced as control cells for counter selection. We further affirmed U2 and U8 identified EGFRvIII on the surface of target cells specifically. Then we radiolabeled U2 with 188Re to serve as a molecular imaging probe and observed 188Re -labeled U2 significantly targeted EGFRvIII over-expressing glioblastoma exnografts in mice. In conclusion, aptamers obtained from whole cell-SELEX strategy have great potential as molecular imaging probes that are probably beneficial to GBM diagnoses.

## Introduction

Glioblastoma (GBM) is the most common and malignant tumor of the central nervous system with a poor overall survival of 12–14 months upon diagnosis [1], [2]. Genetic alterations such as epidermal growth factor receptor (EGFR) gene amplification and mutation are main drivers promoting GBM progression and malignancy [3]. The most common mutant of EGFR, EGFRvIII, which results from a deletion of the extracellular amino acids 6 to 273 (exons 2 to 7), occurs at an overall frequency of 25–64% when assessed by multiple techniques in GBM [4] and contributes to constitutively active oncogenic signaling that correlates with worse prognosis [5]. Its increased expression may influence multiple aspects of tumor biology, including survival, proliferation, motility and invasiveness, and resistance to treatment [6]–[8].

Early detection of cancer and its metastasis can dramatically change treatment and improve prognosis. As the importance of EGFRvIII for GBM, imaging the status of EGFRvIII could be of great value in stratifying patients and monitoring treatment in GBM. Molecular imaging, using specific molecular probes to visualize, characterize and measure biological processes at the molecular and cellular levels in humans and other living systems [9], plays a more and more important role in early diagnosis of various diseases, especially cancer, over the last decade. With many advantages over other commonly used targeting ligands (e.g. antibodies) such as easier synthesis, better thermal stability, smaller size, lower immunogenicity, more versatile chemistry [10], aptamers becomes promising molecular imaging probes in the molecular imaging field. Aptamers are single-stranded oligonucleotides with excellent affinity and specificity for target recognition and evolved from random oligonucleotide libraries by a process called systematic evolution of ligands by exponential enrichment (SELEX). The recently developed cell-based aptamers selection, termed cell-SELEX, uses intact living cancer cells as target and closely related cells as control to produce aptamers capable of identifying molecular differences between cancer cells [11]. Until now, a variety of aptamers which are against different caner cells have been isolated from whole cell-SELEX, only a few of them have been tested for in vivo imaging [12], [13].

Because of the superb sensitivity and clinical applicability of PET and SPECT imaging, development of radiotracers for imaging EGFR and its mutation has attracted intense interest. Among a number of positron-emitting nuclides, ^188^Re is an interesting option for labeling molecular probes and pharmaceuticals by virtue of its favorable physical and nuclear properties [14]–[17]. ^188^Re emits high energy beta rays which are suitable for radiotherapy and its decay is accompanied by a 155 keV predominant energy γ-emission for imaging, biodistribution, or absorbed radiation dose studies [18].

Herein we have performed a whole cell-SELEX procedure, during which a glioma U87MG cell line with EGFRvIII overexpression (U87-EGFRvIII) is used as target cells and U87MG cell line is used as control cells, and generated four highly specific DNA aptamers able to distinguish U87-EGFRvIII cells from U87MG cells, two of which (named U2 and U8) bound at high affinity to EGFRvIII on the surface of target cells. Subsequently we have adopted ^188^Re to radiolabel U2 and ^188^Re-labeled U2 significantly accumulated in the EGFRvIII over-expressing glioblastoma exnografts in nude mice, revealing promising potential of U2 to be a superior molecular probe for glioblastoma imaging and diagnoses.

## Materials and Methods

### Ethics statement

All animal work was conducted according to protocols and regulations approved by Laboratory Animal Ethics Committee of Southern Medical University (permit number: 2012-041).

### Cell culture and tumor model

U87MG cell line was maintained in Dulbecco's modified Eagle's medium (DMEM) supplement with 10% fetal bovine serum (FBS), 100 U/ml penicillin and 100 ug/ml streptomycin (P/S). U87-EGFRvIII cell line [19] (obtained from Dr. Webster Cavenee, Ludwig Cancer Institute, San Diego, CA) was grown in DMEM supplement with 10% FBS, 1% P/S and 100 mg/ml Geneticin. All cells were cultured at 37°C in a 5% CO_2_/95% air atmosphere.

Male BALB/c nude mice at 4–6 weeks of age were supplied by Laboratory Animal Centre, Southern Medical University. 2×10^6^ U87-EGFRvIII cells resuspended in 100 ul of modified RPMI-1640 culture medium were injected subcutaneously into the right rump of each nude mouse. Formation of tumors was observed two weeks later.

### Cell-SELEX procedure

A random primary DNA library, termed GN, containing a central random region of 40 nucleotides was subjected to cell-SELEX process [11]. 5′-ATCCAGAGTGACGCAGCA(N40)TGGACACGGTGGCTTAGT-3′

The 5′ FITC-labeled sense primer:5′-FITC-ATCCAGAGTGACGCAGCA-3′

The 5′ biotin-labeled antisense primer:5′-biotin-ACTAAGCCACCGTGTCCA-3′

The cell-SELEX strategy was performed as described [11] with a few modifications. DNA library was heated at 95°C for 5 min and snap–cooled on ice for 5 min alone, then placed at room temperature for 30 min in binding buffer (0.45 g of glucose, 10 mg yeast tRNA, 5 mg salmon sperm DNA, 0.1 g BSA and 0.5 ml of 1 M MgCl_2_ in 100 ml of 1×phosphate-buffered saline [PBS]) before incubated with cells. After incubation, protease K was added to the washed adherent cells at 37°C for 15 min to detach cells. Since the fourth round, the generated ssDNA pool was first incubated with U87MG cells for counter selection and unbound sequences were then incubated with U87-EGFRvIII cells.

To increase the stringency of selection, the volume of DNA pool and incubation time were gradually decreased, while the number and time of washings were increased during selection process (Table 1).

**Table 1:** Changes of selection conditions in cell-SELEX process.

Cycle	Cell density	ssDNA (pmol)	Yeast tRNA (ug/uL)	Salmon sperm DNA (ug/uL)	Incubation time	Washing conditions
1	2×10^6^	1000	0.1	0.05	60 min	3×1 min
2	1×10^6^	800	0.1	0.1	40 min	3×2 min
3	4×10^5^	200	0.1	0.1	40 min	3×3 min
4*	4×10^5^	150	0.1	0.1	40 min	3×3 min
5*	4×10^5^	150	0.5	0.1	30 min	4×2 min
6*	4×10^5^	100	1	1	30 min	4×2 min
7*	4×10^5^	80	0.5	1	30 min	5×2 min
8*	4×10^5^	100	1	1	30 min	5×2 min
9*	2×10^5^	100	1	1	30 min	5×2 min
10*	2×10^5^	100	1	1	30 min	5×2 min
11*	2×10^5^	100	1	1	30 min	5×2 min

*U87MG cells were introduced for counter selection since the 4^th^ round.

### Preparation of ssDNA by streptavidin-coated magnetic beads purification followed by alkaline separation

A filter was inserted into the bottom of a DNA synthesis column. 1.5 ml of streptavidin-coated magnetic beads (Promega, Madison, WI) suspension was added, washed with 1× DPBS. 300 ul of PCR product was passed through the column five times with a speed of 2 drops/3 seconds controlled by a syringe without a needle. 400 ul of 0.2 M NaOH was added into the washed beads to split dsDNA. FITC-labeled ssDNA was collected and then precipitated by addition of 10 mM MgCl_2_, 0.3 M NaAc and 2.5-fold of ethanol by volume at −20°C over night. After centrifugation, obtained pellet was rinsed with 70% ethanol and dried at room temperature (RT).

### Flow cytometry binding assay

200 pmol each of FITC-labeled aptamers and GN were incubated with 5×10^5^ U87-EGFRvIII and U87MG cells dissociated with nonenzymatic dissociation buffer on ice for 40 min, respectively. Samples were washed three times and resuspended in 1×PBS. Fluorescence intensity was determined by counting 10,000 events in a FACScan cytometer (Becton Dickinson, Franklin Lakes, NJ). Experimental data were analyzed with Flowjo7.6.1 software (TreeStar Inc, Ashland, USA).

### Enzyme-linked immunosorbent assay (ELISA)

U87-EGFRvIII cells (2×10^4^ cells per well) were plated in 96-well plates, fixed with 4% paraformaldehyde for 15 min, blocked with 3% BSA in PBST (0.01% Tween 20 in 1× PBS, pH 7.4) at 37°C for 40 min, washed with PBST and incubated with various concentrations of biotinylated aptamers at 37°C for 40 min. 50 ul of strepavidin-conjugated horseradish peroxidase (1∶1000 in PBS; Sigma, St. Louis, MO) was added into each well and left to incubate for 30 min at 37°C. 50 ul of TMB solution was added and further incubated for 30 min. The reaction was terminated with 25 ul of 2M H_2_SO_4_ and absorbance was measured at 450 nm. Apparent equilibrium dissociation constants (*K*_d_) values for each aptamer were determined by nonlinear regression according to the equation: Y = B_max_*X/(K_d_+X) using SigmaPlot 12.0.

### Protease K digestion assay

The washed 5×10^5^ U87-EGFRvIII cells were dissociated with protease K at 37°C for 3 min and 10 min respectively. Further action was halted by addition of FBS. After washing, samples were incubated with FITC-labeled aptamers and then subjected to flow cytometry analysis.

### Pull-down assay

RIPA buffer containing 1% (v/v) complete protease inhibitor cocktail was added into U87-EGFRvIII and U87MG cells. Samples were centrifuged and supernatant was collected. Total protein levels were determined using the BCA Protein Assay Reagent kit. Streptavidin beads were incubated with 300 pmol each of biotin-labeled U2, U8 and GN at 37 °C for 30 min. The washed bead-aptamer mixtures were incubated with 600 ug cell extracts at 37 °C for 1 h. After washing, the mixtures were heated at 100 °C for 10 min in 50 ul of 2×sodium dodecyl sulfatepolyacrylamide gel electrophoresis (SDS-PAGE) sample buffer and then resolved on immunoblotting analysis. The primary antibody used was anti-EGFR (Santa Cruz, California, USA).

### Direct radiolabeling of aptamer with ^188^Re

10 ug U2 or GN was mixed with 10 ul of 0.2 M acetic acid (PH 5.0), 50 ul of 3 mg/0.1 ml SnCl2, 100 ul of 10 mg/0.1 ml ascorbic acid. Subsequently, 0.6 ml of 0.672 mCi/0.3 ml ^188^Re eluent was added and warmed at 37 °C for 1.5 h. The labelled aptamer was purified by C18-Waters Sep-Pak reverse-phase column and radiochemical purity was determined by paper chromatogram using 70% acetone, saline solution, and a mixture of 30% ethanol: ammonia water: water (2∶1∶5) as solvents respectively, on 1×17.5 cm strips of Xinhua No. 1 paper. Radioactivity was measured in a scintillation γ-counter (USTC ZONKIA, Anhui, China).

### *In vivo* and *ex vivo* organ imaging of tumor-bearing mice

Mice with U87-EGFRvIIIf xenografts were intravenously injected with 300 ul of ^188^Re-aptamer complexes via the tail vein or intratumorally injected with 200 ul of complexes directly. Then mice were imaged in vivo using single photon emission computed tomography (SPECT, GE Medical Systems Is.I, Isael) 1 h after tail veil injection, 0.5 h and 3 h after intratumor injection respectively. Finally, 3 h after tail vein injection the animals were sacrificed. The organs were dissected and imaged with SPECT.

### Statistic analysis

Statistical analysis was performed by One-Way ANOVA for analysis of multiple groups or Independent-Samples T Test for analysis of two groups under SPSS 13.0 program. *K*_d_ values for each aptamer were calculated by nonlinear regression using SigmaPlot 12.0 software.

## Results

### Selection of aptamers

**Figure 1:**
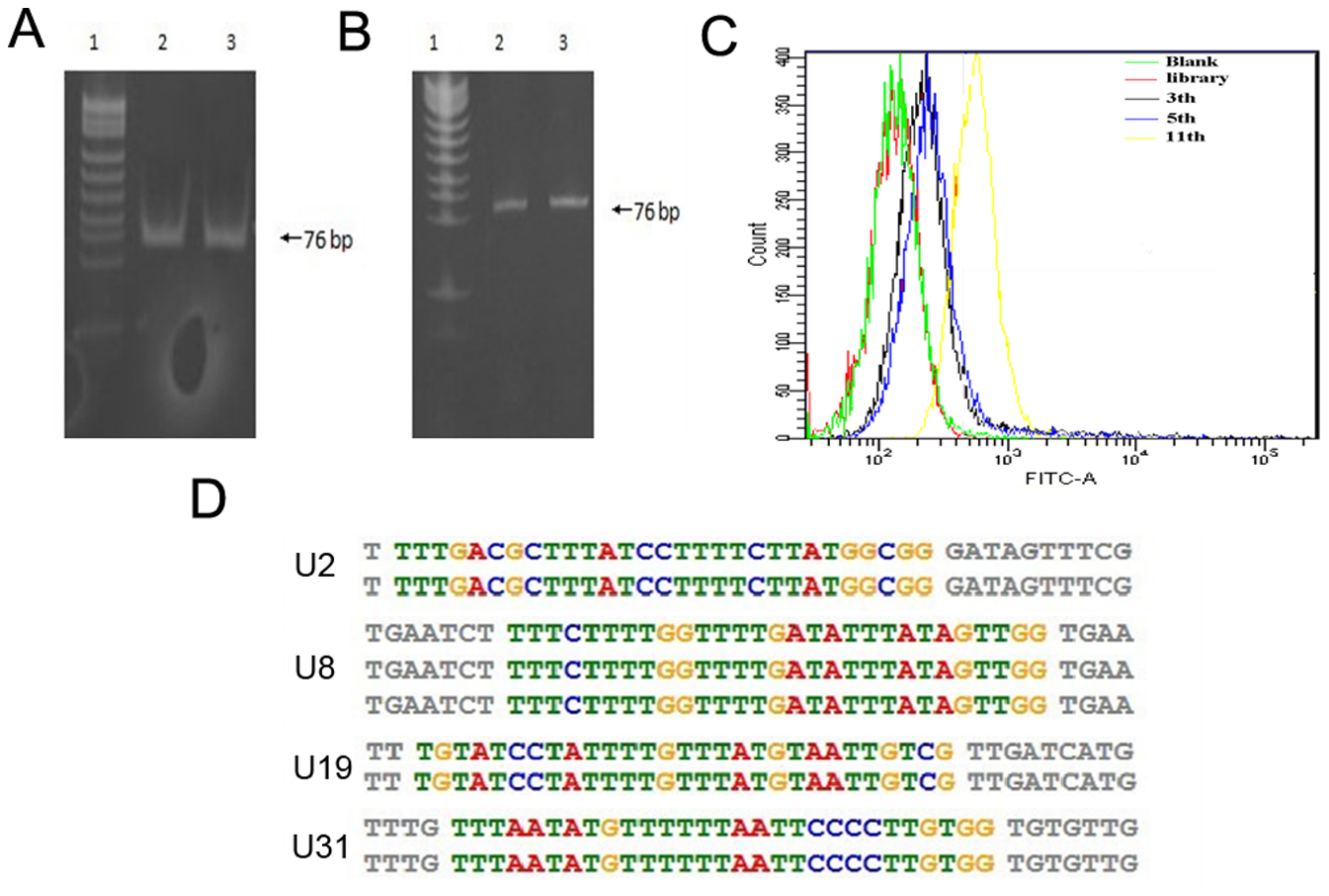
Generation of aptamers by whole cell -SELEX process. (A) DsDNA amplified by PCR during selection process was identified by 10% native polyacrylamide gel electrophoresis. Lane 1: pUC18DNA/Mspl/Marker. Lane 2, 3: dsDNA. (B) 7 M urea 8% denatured polyacrylamide gel electrophoresis was used to confirm obtained ssDNA at the end of each round. Lane 1: pUC18DNA/Mspl/Marker. Lane2: initial DNA pool GN. Lane 3: ssDNA. (C) Enrichment of selected pools was monitored by flow cytometry. FITC-labeled GN (**red**), the 3^rd^ round ssDNA pools (**black**), the 5^th^ round ssDNA pools (**blue**), the 11^th^ round ssDNA pools (**yellow**) were incubated with U87-EGFRvIII cells respectively, then fluorescence intensity was detected. Unlabeled U87-EGFRvIII cells (**green**) were used as blank control. (D) Sequence homology analysis by MEME online software.

During each round of selection, dsDNA obtained by PCR amplification was identified as a single band with a molecular weight of 76 base pairs on 10% native polyacrylamide gel (Fig. 1A). Affinity purification on streptavidin-coated magnetic beads followed alkaline separation was performed to get FITC-labeled ssDNA, which was confirmed by 7 M urea 8% denatured polyacrylamide gel electrophoresis (Fig. 1B), for next round of selection. The enrichment of the selected pools, resulting in the evolution of potential aptamer candidates, was monitored by flow cytometry. FITC-labeled GN and ssDNA pools of the 3^rd^, 5^th^, 11^th^ round were incubated with U87-EGFRvIII cells respectively. Significant fluorescence signal intensity shift from the 5^th^ to the 11^th^ round was observed, indicated an obvious enrichment in round 11 (Fig. 1C). The ssDNA generated from the 11^th^ round was cloned and grouped in families based on their primary sequence similarity. We identified four families of highly related aptamers with a great amount of thymine repetition (Fig. 1D) and selected four sequences (one sequence from each family) as possible aptamer candidates (Table 2).

**Table 2:** *K*_d_ value of the selected aptamers.

Name	Sequence	*Kd*(nM)
U2	ATCCAGAGTGACGCAGCATTTTGACGCTTTATCCTTTT CTTATGGCGGGATAGTTTCG TGGACACGGTGGCTTAGT	3.37±0.98
U8	ATCCAGAGTGACGCAGCATGAATCTTTTCTTTTGGTTT TGATATTTATAGTTGGTGAA TGGACACGGTGGCTTAGT	4.35±1.17
U19	ATCCAGAGTGACGCAGCATTTGTATCCTATTTTGTTTA TGTAATTGTCGTTGATCATG TGGACACGGTGGCTTAGT	16.78±5.90
U31	ATCCAGAGTGACGCAGCATTTGTTTAATATGTTTTTTA ATTCCCCTTGTGGTGTGTTG TGGACACGGTGGCTTAGT	8.10±2.36

Michaelis-Menten binding curves to evaluate *K*_d_ (nM) were performed as described in Materials and Methods; standard deviation values were determined from three independent experiments.

### Selected aptamers specifically bind to U87-EGFRvIII cells with high affinity

**Figure 2:**
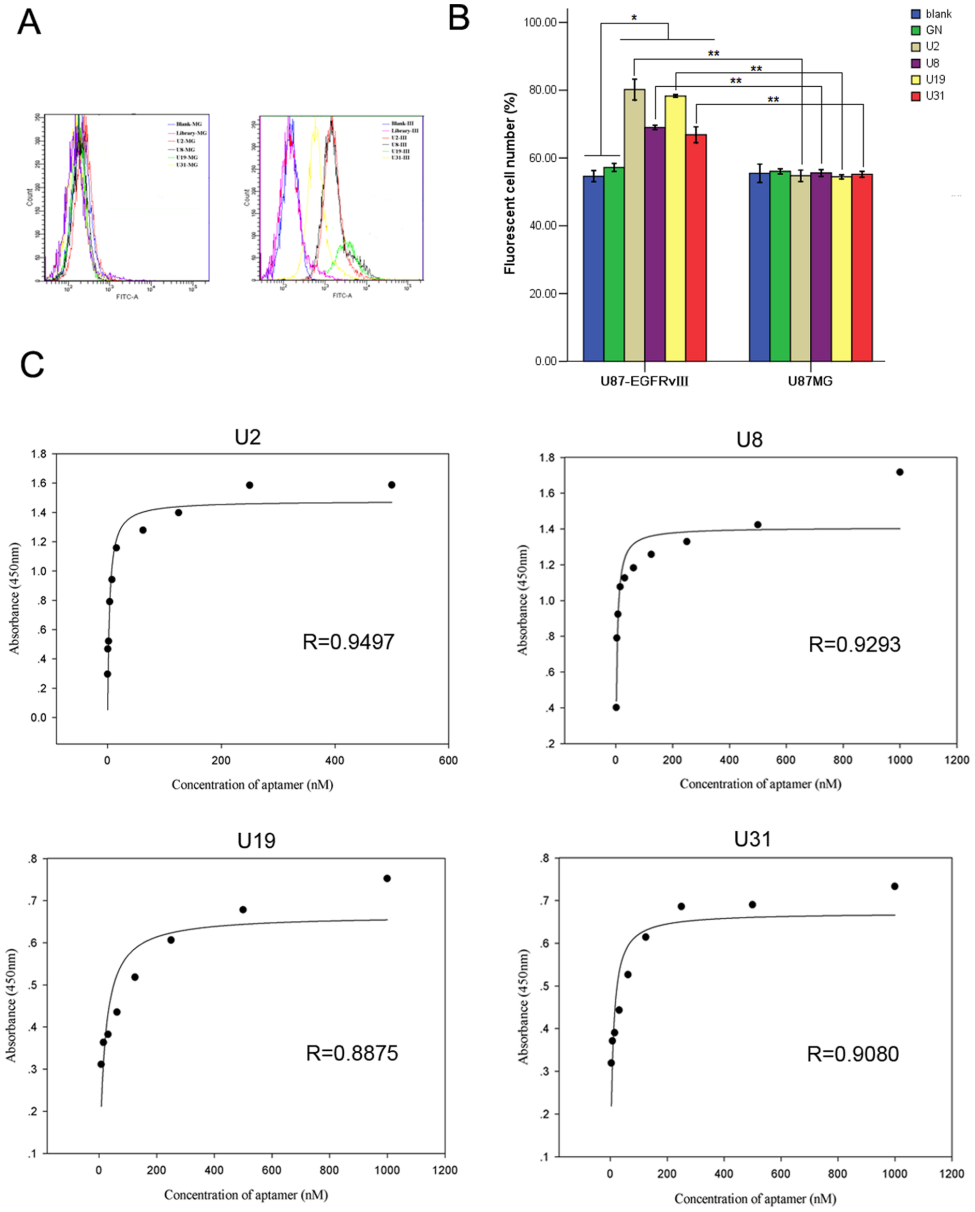
Good specificity and high affinity of selected aptamers to U87-EGFRvIII cells. (A) FITC-labeled aptamers U2 (**red**), U8 (**black**), U19 (**green**), U31 (**yellow**), GN (**pink**) were incubated with U87-EGFRvIII cells (right) and U87MG cells (left), respectively, then subjected to flow cytomety. Unlabeled cells (**blue**) were used as blank control. (B) Changes in cell number with detectable fluorescence reflected fluorescence intensity shift. Data represent mean ± SD of three independent experiments. * *P*<0.05, ** *P*<0.01. (C) Binding curve of U2, U8, U19 and U31 on U87-EGFRvIII cells. The mean absorbance from three independent experiments for each aptamer concentration was used to plot the binding curve.

We wondered whether the four selected aptamers (U2, U8, U19 and U31) can efficiently target U87-EGFRvIII cells. To this aim we conducted flow cytometry binding assay. The fluorescence shift of FITC-aptamers binding to U87-EGFRvIII cells demonstrated all four aptamers bound specifically to U87-EGFRvIII cells and had no specific binding to U87MG cells. GN did not display binding to both cell lines (Fig. 2A, B). ELISA showed these aptamers bound at high affinity to U87-EGFRvIII cells, with *K*_d_ ranging between 3.37 nM and 16.78 nM (Fig. 2C, Table 2).

### Aptamers U2 and U8 specifically target EGFRvIII

**Figure 3:**
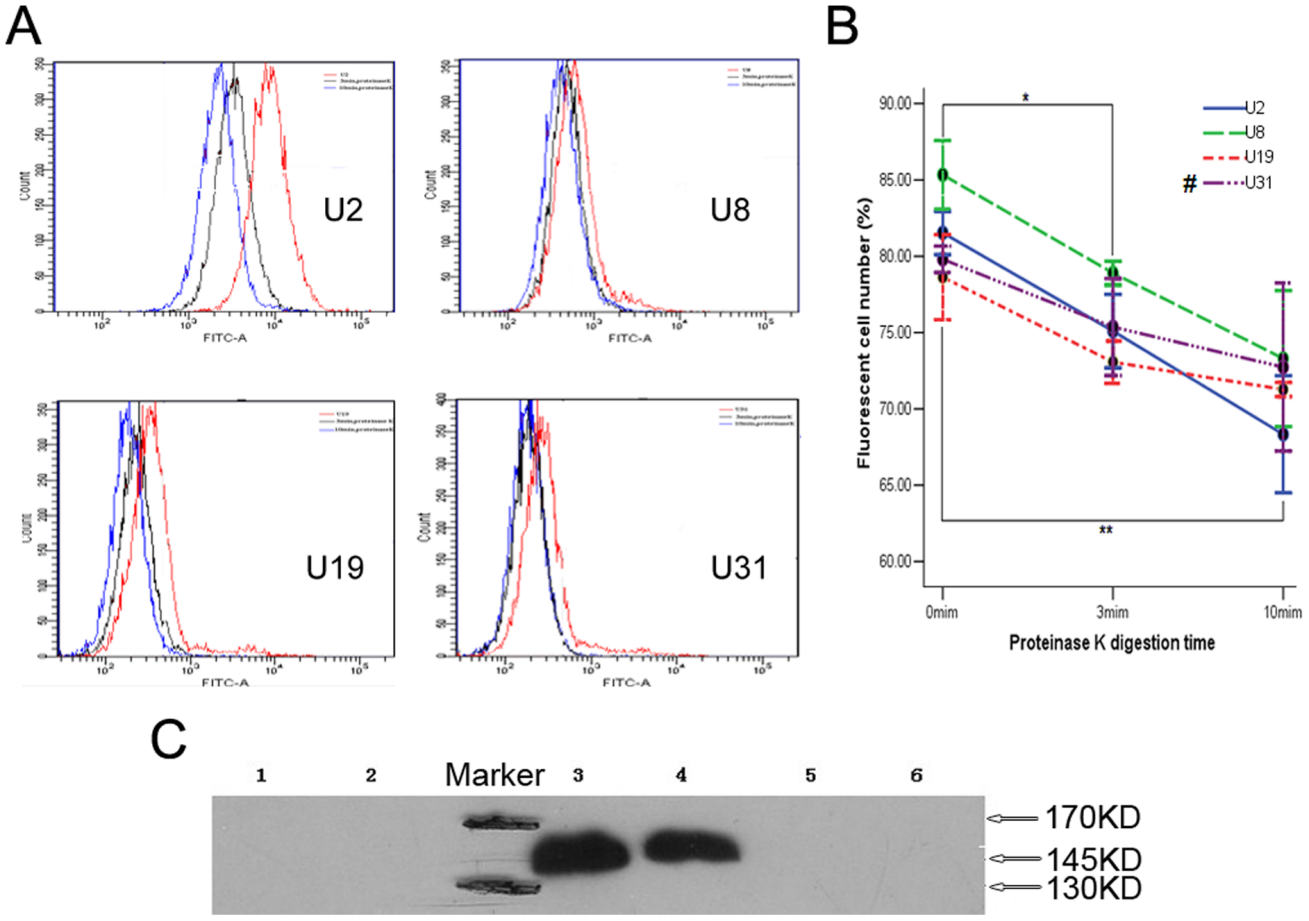
Aptamers U2 and U8 target EGFRvIII specifically. (A) As protease K digestion time increasing from 3 min (**black**) to 10 min (**blue**), transition of the fluorescence intensity shift to the left illustrated a signal intensity decrease. The binding of FITC-labeled aptamers to intact U87-EGFRvIII cells (0 min) was used as positive control (**red**). (B) Changes in cell number with fluorescence as digestion time increasing. Data represent mean ± SD of three independent experiments. * *P*<0.05, ** *P*<0.01. #: There was no statistical difference at different time points in U31 group. (C) The specific interaction of U2 and U8 with EGFRvIII was determined by affinity purification on streptavidin beads of cell lysate treated with biotin-labeled aptamers followed by immunoblotting with anti-EGFR antibody. EGFRvIII has a molecular weight of 145 kDa compared with that of 170 kDa for EGFRwt. Obvious bands at 145 kDa indicated the spicific target of aptamers was EGFRvIII. Lanes (left to right): 1, GN with U87MG cell lysate; 2, GN with U87-EGFRvIII cell lysate; 3, U2 with U87-EGFRvIII cell lysate; 4, U8 with U87-EGFRvIII cell lysate; 5, U2 with U87MG cell lysate; 6, U8 with U87MG cell lysate. Three independent experiments were performed.

To investigate whether the selected aptamers interacted with certain surface protein on U87-EGFRvIII cells, protease K digestion assay was performed. The binding of these aptamers, especially of U2, to U87-EGFRvIII cells was decreased as digestion time increased (Fig. 3A, B), suggested aptamers did bind to specific surface protein on target cells. We further verified that U2 and U8 specifically interacted with EGFRvIII on U87-EGFRvIII cell surface, whereas no binding was obtained with GN by pull-down assay (Fig. 3C).

### ^188^Re-U2 complex specifically targets U87-EGFRvIII xenograft tumor in mice

**Figure 4:**
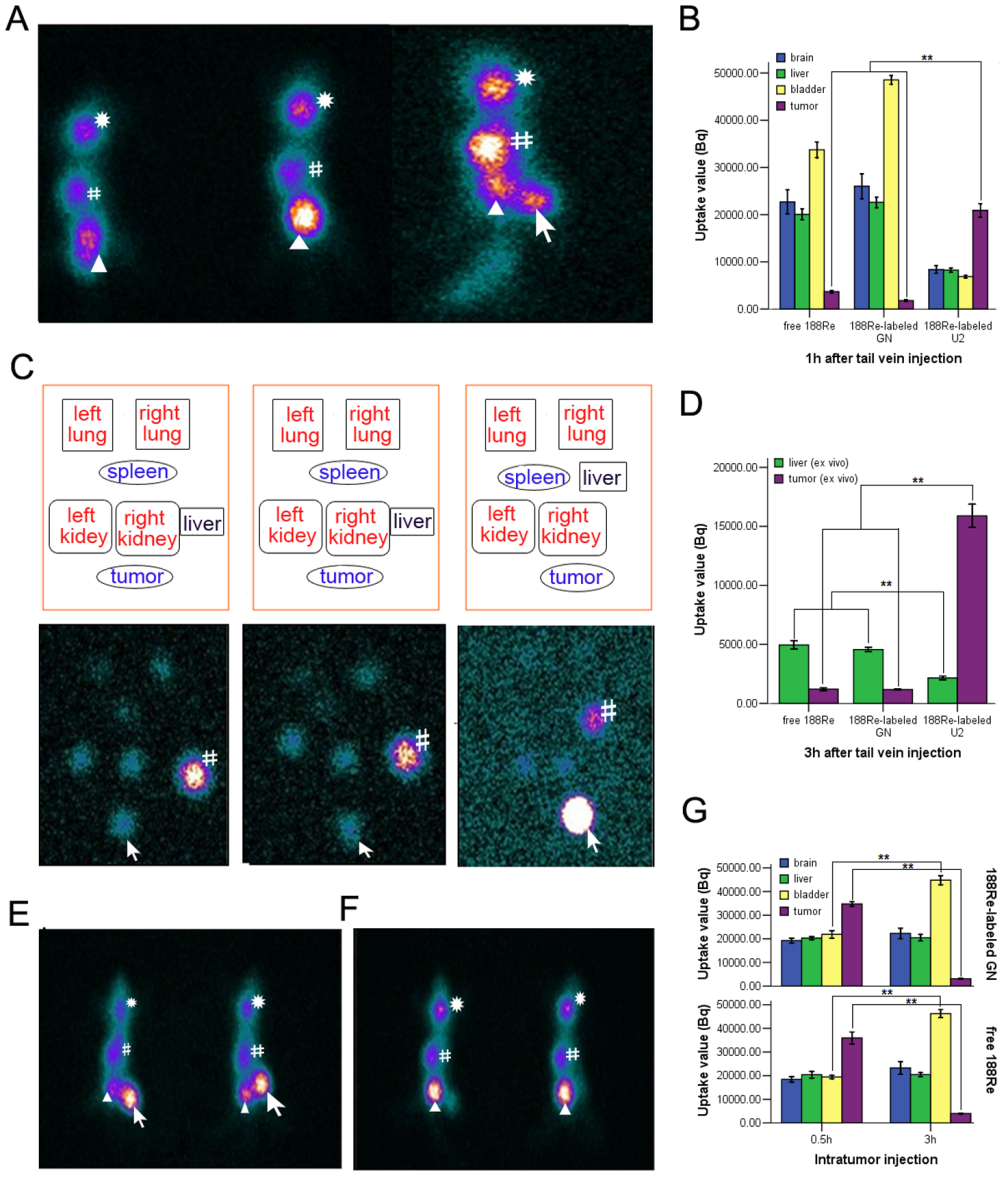
^188^Re-labeled U2 imaged exnografts glioblastoma in mice successfully. (A) Imaging of mice by SPECT 1 h after tail vein injection of free ^188^Re (left), ^188^Re-labeled GN (middle) and ^188^Re-labeled U2 (right). (B) Relative uptake values of the organs were counted and quantified using VG Acq data acquisition and processing system from SPECT. In (B), chart showed comparison of uptake values among different groups at 1 h after tail vein injection. (C) Up: Placement of real ex vivo organs. Down: Corresponding imaging of ex vivo organs by SPECT 3 h after tail vein injection of free ^188^Re (left), ^188^Re-labeled GN (middle) and^188^Re-labeled U2 (right). (D) Comparison of uptake values in ex vivo organs at 3 h after tail vein injection. (E–F) Imaging of mice 0.5 h (E) and 3 h (F) after intratumor injection of free ^188^Re (left), ^188^Re-labeled GN (right). (G) Changes of uptake values at 0.5 h and 3 h after intratumor injection. Asterisk indicated brain; pound  =  liver; triangle  =  bladder; arrow  =  tumor. Error bars depict means ± SD (n = 3). ** *P*<0.01.

Aptamer U2, with a *K*_d_ value of 3.37±0.98 nM was a slightly stronger binder than other three aptamers, was chosen for tumor imaging investigation. We successfully radiolabeled U2 and GN (as a control) with ^188^Re and the labelling efficiency was 68% and 70% respectively. Average radiochemical purity (RCP) was >60%. SPECT revealed 1 h after tail vein injection ^188^Re-labeled U2 can be detected in tumor tissue, in contrast no ^188^Re-labeled GN and free ^188^Re were detectable intratumorally (Fig. 4A, B). 3 h after tail vein injection in ex vivo organ SPECT ^188^Re-labeled U2 showed an excellent tumor uptake while ^188^Re-labeled GN and free ^188^Re mainly accumulated in liver, indicating the capability of U2 to target U87-EGFRvIII xenograft tumor efficiently (Fig. 4C, D). On the other hand, ^188^Re-labeled GN and free ^188^Re retained in tumor 0.5 h after intratumor injection and disappeared 3 h later, accompanied by appearances of high density image in the bladder, which implied the major route of excretion of these ^188^Re-labeled aptamers may be via the urinary tract (Fig. 4E–G).

## Discussion

We conducted whole cell-SELEX technology to select DNA aptamers against U87-EGFRvIII cells. The introduction of U87MG cells as control cells for negative selection can prevent generation of common sequences target both cell lines instead of unique surface receptor present on U87-EGFRvIII cells. As expected, the selected aptamers bound to U87-EGFRvIII cells with good specificity. The low *K*_d_ values of these aptamer for U87-EGFRvIII cells determined by ELISA revealed high affinity to their target. Further we were able to testify aptamers U2 and U8, with better binding ability than U19 and U31 as shown in ELISA, bound to EGFRvIII protein specifically.

The significant accumulation of ^188^Re-labeled U2, the brand-new radioactive tracer in U87-EGFRvIII xenografts demonstrates the great potential of U2 as a molecular probe for radionuclide imaging of GBM associated with high levels of EGFRvIII in living body. In respect of whether ^188^Re-labeled U2 can penetrate the blood-brain barrier (BBB) to target brain GBM, we consider incorporating the complex into nanoparticle systems to cross the BBB. Key features of nanoparticles such as versatile composition, unique physical properties, passive targeting abilities, as well as tunable surface functionality for active targeting, enable the transportation of diagnostic or therapeutic agents across the BBB [20]. Another alternative is to employ membrane-permeable peptide carriers, usually oligopeptides that can rapidly cross the plasma membrane, even the BBB and deliver a range of bioactive molecules to the cytoplasm or nucleus, to mediate in vivo delivery of the complex [21]. Some problems such as pharmacokinetics and toxicity of ^188^Re-labeled U2 upon systemic delivery are worthy of detailed research. However, in contrast to large amount of doses necessary for therapeutic purposes, only trace amount of aptamers will be needed for imaging applications, we thus speculate these aspects are not of major concern. Since EGFRvIII is expressed in GBM, non small cell lung carcinomas, and breast carcinomas [4], whether ^188^Re-labeled U2 can image other malignancies with EGFRvIII overexpression remains to be investigated. The enrichment of ^188^Re-labeled GN in liver 3 h after tail vein injection may because liver is an organ with abundant blood flow. But its actual uptake value (4531±7.28 Bq) is much smaller than that of ^188^Re-labeled GN in the bladder 1 h after tail vein injection (48817±67.52 Bq), suggesting the predominant elimination of the radiolabeled aptamers is through urinary excretion, which is confirmed by the accumulation of ^188^Re-labeled GN in the bladder 3 h after intratumor injection and is consistent with previous reports [14], [15].

Some cell-SELEX aptamers have been generated against EGFR in recent years, but few are designed for in vivo imaging. A ^99m^Tc labeled RNA aptamer specific to human epidermal growth factor receptor 2 (HER2) is recently reported to image HER2-overexpressing ovarian cancer [22]. And aptamers used for radionuclide imaging are mostly labeled with ^99m^Tc through a chelator and/or a co-ligand [23]–[25]. Since the types of ligand and chelator used can significantly affect lipophilicity and may alter the biodistribution of radioconjugated oligonucleotides in vivo [26], direct labeling of aptamer may eliminate these effects and improve tumor uptake. This is the first study investigating the use of ^188^Re to directly radiolabel anti-EGFRvIII aptamer for related tumor imaging.

In conclution, aptamers generated by whole cell-SELEX have excellent recognition capability to target cells both in vitro and in vivo, and have showed exceptional biostability and sensitivity for radionuclide imaging. Early diagnosis of malignant tumor depends on specific molecular probes. The fact that ^188^Re-labeled U2 has been successfully applied as a target probe for in vivo GBM imaging prompts potential application to diagnoses of GBM.

In addition, because of the radiopharmaceutical characteristic of ^188^Re and specific interaction between U2 and EGFRvIII, it cannot be excluded that ^188^Re-labeled U2 or U2 alone possesses the capability to inhibit EGFRvIII over-expressing GBM growth directly. Related work is under way.
